# The myopia of crowds: Cognitive load and collective evaluation of answers on Stack Exchange

**DOI:** 10.1371/journal.pone.0173610

**Published:** 2017-03-16

**Authors:** Keith Burghardt, Emanuel F. Alsina, Michelle Girvan, William Rand, Kristina Lerman

**Affiliations:** 1 Dept of Computer Science, University of California at Davis, Davis, CA, United States of America; 2 Dept of Political Science, University of California at Davis, Davis, CA, United States of America; 3 University of Modena and Reggio Emilia, Modena MO, Italy; 4 Dept of Physics, University of Maryland, College Park, MD, United States of America; 5 Santa Fe Institute, Santa Fe, NM, United States of America; 6 Department of Business Management, North Carolina State University, Raleigh, NC, United States of America; 7 Information Sciences Institute, University of Southern California, Marina del Rey, CA, United States of America; Consejo Nacional de Investigaciones Cientificas y Tecnicas, ARGENTINA

## Abstract

Crowds can often make better decisions than individuals or small groups of experts by leveraging their ability to aggregate diverse information. Question answering sites, such as Stack Exchange, rely on the “wisdom of crowds” effect to identify the best answers to questions asked by users. We analyze data from 250 communities on the Stack Exchange network to pinpoint factors affecting which answers are chosen as the best answers. Our results suggest that, rather than evaluate all available answers to a question, users rely on simple cognitive heuristics to choose an answer to vote for or accept. These cognitive heuristics are linked to an answer’s salience, such as the order in which it is listed and how much screen space it occupies. While askers appear to depend on heuristics to a greater extent than voters when choosing an answer to accept as the most helpful one, voters use acceptance itself as a heuristic, and they are more likely to choose the answer after it has been accepted than before that answer was accepted. These heuristics become more important in explaining and predicting behavior as the number of available answers to a question increases. Our findings suggest that crowd judgments may become less reliable as the number of answers grows.

## 1 Introduction

Are crowds wiser than informed individuals? Generally speaking, a crowd’s collective opinion—whether through ballots, votes, likes, or thumbs up and down—is often used to help identify quality items and rank-order them in online systems, which determines how much attention items receive [1], as well as users’ incentives for participating [2]. The observation that collective opinions can outperform individual experts even when the crowd is less-informed than the experts [3] serves as a basis for many “wisdom of crowds” applications [4–6] and even a motivation for juries in trials [7]. Recent evidence, however, suggests that the collective decisions of the crowd are not foolproof. One known limitation is social influence, which biases individual judgments and degrades crowd performance [8], obscuring the underlying quality of choices [9]. Moreover, competition for recognition in crowdsourced evaluation may lead to a greater diversity of ideas, but also a greater rejection rate of high quality ideas [10]. In this paper we identify other factors that can potentially degrade crowd wisdom—namely, heuristics and cognitive load—and study their impact on a common crowdsourcing application: question answering.

We carry out an empirical study of Stack Exchange (http://stackexchange.com), a network of more than two hundred question answering (Q&A) communities, where millions of people post questions on a variety of topics, and others answer them asynchronously. Like other Q&A sites, such as Quora and Yahoo! Answers, Stack Exchange has a number of features for enhancing collaborative knowledge creation. In addition to asking and answering questions, users can evaluate answers by (1) *voting* for them, and (2) askers can *accept* a specific answer to their question. The votes, in aggregate, reflect the crowd’s opinion about the quality of content, and are used by Stack Exchange to surface the most helpful answers. They also provide a lasting value to the community [11], enabling future users to identify the most helpful answers to questions without asking the questions themselves.

We find that the number of answers users have to parse through can dramatically affect their choices. As the number of available answers to a question increases, users appear to rely to a greater extent on simple heuristics, such as answer’s list position, to pick what they consider the best answer, potentially limiting the utility of Q&A boards. In addition, we find that these behavioral biases allow for users to choose answers in an increasingly predictable way, as the number of answers grows, running counter to our intuition that increasing the number of choices makes human decisions less predictable.

Our work also addresses some of the challenges of analyzing heterogeneous data. Large-scale datasets of human behavior, such as this one, provide new opportunities to study decision-making processes in crowdsourcing systems. In contrast to laboratory studies, which typically involve dozens of subjects, behavioral data is collected from millions of people under real-world conditions. Mining observational behavioral data, however, presents significant computational and analytic challenges. Human behavior is noisy and highly diverse: aggregating data to improve the signal-to-noise ratio may obscure underlying patterns in heterogeneous data and even lead to nonsensical conclusions about human behavior [12]. We discover that splitting data by the number of answers addresses one of the more significant sources of heterogeneity, potentially providing greater predictive power in future models.

### Our contributions

We use penalized logistic regression to uncover factors associated with users’ decisions to vote for or accept answers on all Stack Exchange communities. To partly control for heterogeneity, we split data by community type and the number of answers each voter sees, and leave out the largest community in our training data to check the robustness of results. By community type we mean technical, programming-related communities (e.g., Ubuntu), non-technical (e.g., cooking), and meta communities that discuss aspects of a particular board itself rather than a particular topic. Parsing data in this way reveals significant differences in the importance of particular attributes, and the predictability of user behavior.

We find that only a few answer attributes are strongly associated with users’ choices, such as the order in which the answer appears and whether it has been accepted by the asker. Users appear to rely on simple heuristics to choose an answer based on its rank, how much screen space it occupies, or whether it was approved by others. These heuristics may be useful proxies for answer quality, but our work suggests otherwise. For example, voters are more likely to choose an answer after it has been accepted than before. Although answer acceptance is often viewed as a standard of answer quality [13–15], the only discernable difference in an answer after acceptance is a signal that the asker chose this answer, suggesting users view acceptance as a useful signal of quality, but are less able to discern that quality on their own.

Moreover, as the number of answers to a question grows, the importance of these attributes in describing behavior increases. Two different explanations are feasible as to why the number of answers a user sees affects their behavior. First, as the number of available answers grow, users may become less willing to thoroughly evaluate all answers, instead increasingly relying on heuristics when choosing an answer to vote for or accept. A similar effect exists in other domains. For instance, information overload impacts consumer’s choice of products [16] and the spread of information in online social networks [17, 18]. An alternative explanation is that later voters are different and happen to rely more on heuristics compared to people who vote early. This view is supported by the observation that users who answer early in a question’s life cycle on Stack Overflow, a programming-related community on Stack Exchange, have a higher reputation than users who answer later [11]; therefore, time acts as a potential source of heterogeneity. In either case, the finding that voters appear to rely on heuristics to a greater extent as the number of answers grows suggests limits of the “wisdom of crowds” on Stack Exchange: a crowd’s judgments become less reliable as proxies of quality as answers to a question accumulate.

The rest of the paper is as follows. In the related work section, we review work related to our current analysis, while, in the materials and methods section, we discuss our data and ways in which we analyze it. Next, in the results section, we discuss our main findings. Finally, in the conclusion section, we review our findings, discuss future work, and discuss ways to improve upon question answering sites.

### Related work

Collective decisions of a group have been claimed to be superior to those of individuals, even better-informed individuals, when the group is large [3, 7], i.e., a crowd. The advantage arises from the group’s ability to aggregate diverse information and thereby, reduce the error [19]. This effect, coined “the wisdom of crowds” [4] is the basis of many online crowdsourcing applications, including question answering [4–6].

Prior research on Q&A sites has shown that a variety of attributes can provide useful insights into content quality [15, 20–22]. For example, Kim and Oh [14] examined how users evaluate information in Yahoo! Answers forums, by examining the comments askers leave on answers. They found socioemotional-, content-, and utility-related criteria are dominant in the choice of the best answer, and found users evaluate information based not only upon the content, but also on cognitive and collaborative aspects. Adamic et al. [23] similarly conducted a large-scale network analysis of Yahoo! Answers forums and found that, for both technical and non-technical sites, answer length and the number of other answers the asker has to choose from are the most significant features to predict the future best answer. One limitation in these previous studies, however, is in assuming that the answer an asker chose was the “best” answer, and did not correct for asker biases, such as user preference towards choosing items at the top of a list, when choosing an answer, which we find can explain user behavior increasingly well.

Several authors [13–15] use logistic regression to determine which attributes best describe high quality answers, and often assume that a “high quality” answer is one an asker accepts, which our work casts doubt on. Other works have examined the impact of speedy answerers on answer quality. Anderson et al. [11] found that early answers in Stack Overflow (the Stack Exchange community that deals with programming questions) tend to be posted by expert users with higher reputation, and subsequent answers come from lower reputation users. While the first answer tends to be more appreciated by the asker, the longer a question goes unanswered, the less likely that an answer will eventually be accepted. Similarly, Rechavi and Rafaeli [24] concluded that askers use response time as a parameter at evaluation time. However, this hypothesis was refuted in other works. Shah [25] analyzed the responsiveness in Yahoo! Answers forums, finding that more than 90% of the questions receive an answer within an hour. However, satisfactory answers may take longer, depending on the difficulty of the question. Interestingly, our work suggests that answer age and chronological order are not particularly important attributes for askers or voters, possibly because high reputation answerers do not strongly affect whether an answer gets voted on or accepted.

In contrast to many previous studies, our paper examines how user-voting patterns may be affected by various answer attributes. This is an important area of study, because people often use votes as a signal of the best answer to a particular problem. A previous paper deduced a set of possible factors that indicate bias in user voting behavior [26]. They provided a method to calibrate the votes inside Q&A sites, based in part on the average value of the answer and the average vote received in the answerer history. This type of calibration is useful to restrict the effects of users who are trying to game the system, or to signal the reputation of answerers. Our work, however, answers a different set of questions: we want to find the role heuristics play in answer evaluation, how voter and asker behaviors differ, and what drives heterogeneity within voter and asker populations. The role of heuristics in human decisions has been studied by behavioral economics [27, 28], but, to the best of our knowledge, our work is the first that investigates the potential impact of heuristics on the performance of crowdsourcing systems.

## 2 Materials and methods

The first Stack Exchange Q&A community, Stack Overflow, was launched in 2008 to answer computer programming questions. Over time, Stack Exchange has added more communities covering several diverse topics, including, as of late 2014 when our data was collected:

- 49 *Technical* communities, which discuss problems relating to computers, such as coding, information security, and Unix-based operating systems;
- 33 *Culture and recreation* communities, which discuss free-time activities, such as learning the english language, bicycles, and anime;
- 17 *Life and Arts* communities, which discuss topics such as cooking, photography, and movies;
- 16 *Science* communities, which discuss questions relating to academic pursuits, such as computer science, mathematics, and statistics; and
- 4 *Business* communities, which discuss Bitcoins, project management, patents, and finance.

There is a *meta* board for each community, in which users discuss the workings and policies of the community, e.g., in Meta Stack Overflow, users discuss the policies of Stack Overflow rather than computer programming itself. Posts that are overly subjective, argumentative, or likely to generate discussion rather than answers, are removed from the website.

A user can post a question, which may receive multiple answers from different people. The asker can *accept* an answer, which generally signifies that the asker finds it helpful, and this signal is displayed prominently. Other users, with a high enough reputation can *vote* an answer up (or down) if they think that it provides helpful (or irrelevant) information. By upvoting more helpful answers, a community collectively curates the information for both the asker and future users interested in the same topic. The difference between the up and down votes is the displayed *score* of the answer. Answers with higher scores are shown, by default, at the top of the web page, so that they are easier to find (answers with the same score are shown in random order). Other attributes, such as an answerer’s reputation, are displayed as well, prompting us to model how it may affect user behavior.

For our study we used data of all user contributions to Stack Exchange from August 2009 until September 2014 (https://archive.org/details/stackexchange, CC BY-SA 3.0 https://creativecommons.org/licenses/by-sa/3.0/, no changes made to the original data), split between training data (before January 1, 2014) and testing data. The data includes 8.56 × 10^6^, 7.84 × 10^5^, and 1.24 × 10^5^ questions for technical, non-technical, and meta boards, respectivly, with, on average 1.5 − 1.85 answers per question, depending on the board type and whether the dataset is for training or testing. In total, there are 2.83 × 10^7^, 5.06 × 10^6^, and 1.30 × 10^6^, votes for the technical, non-technical, and meta boards, respectively, with 11.1%, 21.2%, and 21.8% of the votes in the testing set. In our data, approximately half of the questions have an accepted answer. We focus on these questions in order to study the effect accepting an answer has on voting patterns. For 250 communities, we collect the ID of each post, it’s creation date, the type of post (question or answer), which question an answer is associated with, the date an answer was accepted (if any), and the content of the post. We also recorded vote attributes, including the type of vote (an up- or down-vote), when it was made, and what answer was voted on. Furthermore, we recorded user attributes, such as when a user signed up for a particular board and their reputation at the moment before they posted an answer by applying the rules of Stack Exchange (http://meta.stackexchange.com/how-does-reputation-work) since the very first post was created in 2008 (data is available upon request, and code used to find the reputation and to fit the data can be found here: https://github.com/KeithBurghardt/Stack-Exchange-User-Behavior). Some rules regarding how reputation increases have changed slightly in those periods, which we decided to ignore as an approximation. Furthermore, the reputation bounties were not used to determine reputation because we do not know who gave up their reputation when awarding a bounty. Therefore, reputation calculations will not be completely precise, but we appear to find a close correlation between predicted and actual reputation.

**Fig 1:**
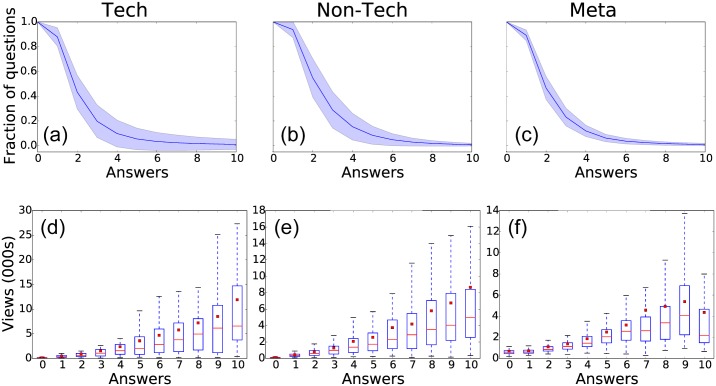
Distribution of the final number of answers to each question and question popularity versus the final number of answers. (Top row) Complementary cumulative distribution of the final number of answers posted in reply to a question as of September, 2014, on (a) technical, (b) non-technical, and (c) meta sites. Shaded areas correspond to the standard deviation in the distributions made for each board. (Bottom row) Number of views per question as a function of the number of answers on (d) technical, (e) non-technical, and (f) meta sites. Boxes indicate 50% confidence intervals, with a red line to indicate the median view count, and a red dot to represent the mean viewcount.

Only the questions that received two or more answers were included in our study (40–50% and 31–41% of the training and testing datasets, respectively). Fig 1 shows the complementary cumulative distribution of the number of answers posted for each question on technical, non-technical, and meta communities for questions as of September, 2014. We see that not only do a large proportion of questions have 2, 3, or more answers, but those with the most answers tend to be the most popular. In other words, determining why one answer is picked as the “best” among many answers becomes an especially important question for the most popular, and presumably most important questions.

### 2.1 Taming heterogeneity

Automatically uncovering homogeneous populations within heterogeneous observational data remains an open research challenge. In our study of Stack Exchange, we use exploratory data analysis to identify potential sources of heterogeneity. For example, users who are interested in technical topics (e.g., programming) may be driven by different factors to contribute to Stack Exchange than those who are interested in non-technical subjects (e.g., cooking), or governance (meta boards). To account for this source of heterogeneity, we split the data by the type of board—technical, non-technical and meta—and run regression analysis separately on each dataset. We further split data by whether the asker eventually accepted an answer in our observation window, how an answer is chosen (vote versus accept), and the number of answers, but find that the greatest source of heterogeneity is the number of answers a question has at the time the user votes for or accepts it.

### 2.2 Logistic regression

Logistic regression is a common model from choice theory for binary decisions [29], because it can be written in the following way:

$$\begin{array}{c} P\left(U,\epsilon \right)=\frac{1}{1+\exp(-U/\epsilon )} \end{array}$$ (1)

where

$$\begin{array}{c} U=\epsilon \beta \cdot X. \end{array}$$ (2)

*U* can be interpreted as the utility of a choice, *ϵ* is a noise parameter to reflect choices being non-deterministic, ***β*** are the regression coefficients, and ***X*** are the answer attributes. Logistic regression can be interpreted as users tending to choose an option if its utility is greater than some threshold, where the probability to choose the option is moderated by noise. We are motivated to use logistic regression to understand the factors driving users to vote for or accept an answer on Stack Exchange, because of its simple theoretical interpretation, although other models, such as random forests [30], could be used as well. Whether other models match data better than the current one is left for future study. The logistic model may still seem an unusual choice, because it assumes users make binary choices, while, in reality, users choose one of several answers available, and therefore could be modeled using multinomial logistic regression. However, the effect the number of answers has on how askers and voters vote in our data seems to directly contradict the independence of irrelevant alternatives (IIA) assumption built into multinomial regression. Although other multi-class models do not depend on the IIA assumption (e.g., [31]), they can be very slow, and it can be difficult to avoid over-fitting without a penalized regression model that we describe below. For all these reasons, logistic regression is the most appropriate model.

Our data is highly multi-dimensional, therefore we use LASSO penalized regression, where parameters are determined by maximizing the likelihood function with the addition of a penalty, to avoid overfitting [32]. The value of this penalty is adjusted to minimize the mean error from ten-fold cross-validation (CV). As a check, we repeated the fits with a different type of penalty—ridge regression—and found the behavior to be qualitatively the same (S1 Fig). The fitting was performed with the R package “glmnet” [33], which allows for fast and accurate determination of regression coefficients, ***β***.

We check the robustness of results by omitting data from the largest community for each board type (technical, non-technical, and meta), and then re-evaluate the regression parameters. The qualitative results were unaffected, and quantitatively, the results were very similar (S2 Fig). For the rest of the paper, we focus on LASSO penalized regressions with all boards included.

### 2.3 Error

The uncertainty in ***β*** (shaded regions in subsequent figures) is found by changing the LASSO penalty term, *λ*, such that

$$\begin{array}{c} \text{min}\left(D\right)\leq D\left(\lambda \right)\leq \text{min}\left(D\right)+\sigma_{\text{min}(D)}, \end{array}$$ (3)

where *D*, the error term in our regressions, is the deviance:

$$\begin{array}{c} D=-2\{\text{log}\left[p\left(y|{\hat{\theta }}_{0}\right)\right]-\text{log}\left[p\left(y|{\hat{\theta }}_{s}\right)\right]\} \end{array}$$ (4)

where $\text{log}[p\left(y|{\hat{\theta }}_{s}\right)]$ is the log-likelihood of the saturated model, with one degree of freedom per observation, while $\text{log}[p\left(y|{\hat{\theta }}_{0}\right)]$ is the log-likelihood for the fitted model. $\text{min}(\hat{\langle D\rangle })$ is the minimum mean deviance, based on ten-fold cross-validation, and *σ*_min(*D*)_ is the standard deviation of this minimum. LASSO regression, like all penalized regression methods, does not have a standard method to calculate uncertainties with high dimensional data [34], therefore we use this method as a reasonable method to extract uncertainties in regression coefficients.

In contrast, the uncertainty in the area under the receiver operating characteristic (ROC) curves are calculated by bootstrapping the pairs of predicted probabilities and outcomes, calculating seperate ROC curve, and the associated area under the curves (AUC). We then use error propogation to determine the AUC error bars when averaged over the number of answers users see. The ROC curve represents the best true positive rate our model can achieve for a given false positive rate, as we vary the discrimination threshold. The AUC, on the other hand, can be interpreted as the average true positive rate across all discrimination thresholds, and is equivalent to the Wilcoxon rank test [35].

### 2.4 Attributes and normalization

We use the following answer attributes in the regression:

1. answerer’s *reputation* at the time the answer was created,
2. mean rate of *reputation increase* over time,
3. answer’s Flesch Reading Ease [36], or *readability*, score,
4. answerer’s *tenure* (i.e., time since joining the site) at the time of the answer,
5. number of *hyperlinks* per answer,
6. binary value denoting whether the *answer was eventually accepted* (for voting only),
7. answer *score* before each vote,
8. default *web page order* for an answer (i.e., its relative position),
9. *chronological order* of an answer (whether it was first, second, third, etc.),
10. *time* since an answer was created, or its age
11. *number of words* per answer,
12. answer’s *word share*, that is the fraction of total words in all answers to the question.

Answerer reputation [13], Flesch readability, and word count [37] were used in previous works as measures of answer quality. We also consider an answer’s rank in the list of answers (what we refer to as web page order) and score, because these variables affect how much attention the answer receives [9, 38, 39], and we empirically find answers are voted on more once accepted (therefore any questions without accepted answers were removed from the study). The other attributes listed above were also examined as additional factors that could affect how answers are voted or accepted. These were, however, not found to significantly affect our results.

There is large variability in attribute values within and across datasets. To account for the variability, we *normalize* all attributes except the web page order and chronological order by mapping them to their associated cumulative distribution function (CDF). CDF normalization is a non-parametric way to reduce the effect of outliers because values are evenly spread between 0 and 1. To test this, we created simulated data with varying distributions and found that CDF normalization creates less variability of regression estimates than normalizing by the standard deviation ($X\to \left(X-\hat{\langle X\rangle }\right)/\hat{\sigma_{X}}$, where $\hat{\langle X\rangle }$ is the estimated mean value of the attribute, and $\hat{\sigma_{X}}$ is the estimated variance), see S3 Fig. Normalization also allows us to compare the relative importance of different attributes via their logistic regression coefficients. We interpret each regression coefficient as the relative effect an increase of an attribute, *X*, by 1% has on the probability to choose that answer. For the web page order and chronological order attributes, we divided by the number of answers available, which is equivalent to a CDF when controlling for the number of answers visible.

When selecting attributes, we made sure correlations with other attributes were reasonably low, and, if they were greater than 0.7, we checked whether removal of the attribute increased the CV error, or reduced the AUC on the tested dataset significantly (see next section). This correlation condition seems very liberal, but we wanted to include as many attributes used in previous literature as possible. To check if this affected our results, we separately removed wordshare, score, whether the answer was eventially accepted, and webpage order, and found results were qualitatively the same.

## 3 Results

We analyze Stack Exchange data to understand what attributes are strongly associated with the decision to vote for an answer or accept it. To do this, we find all attribute values just before an answer was voted for or accepted in our training data, and then estimate attribute coefficients for a logistic regression model. The model was trained on data from August 1, 2009 until December 31, 2013, and then tested on data from January 1 until September 14, 2014.

### 3.1 Answer attributes and behavior

**Fig 2:**
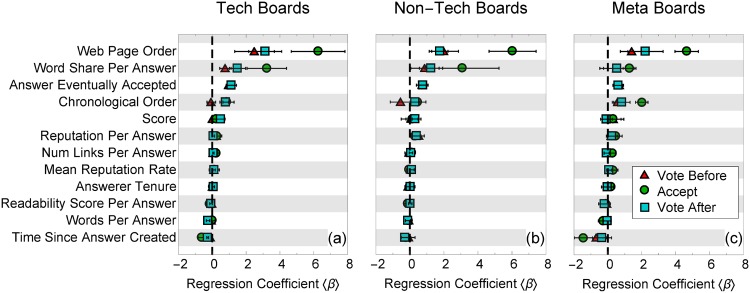
Relative size of regression parameters for answer acceptance and voting. Regression coefficients for answerers to accept (green circles) and voters to vote for an answer both before (red triangles) and after (blue squares) an answer is accepted on (a) technical, (b) non-technical, and (c) meta boards, averaged over the number of available answers from two to twenty. Higher values indicate a stronger relationship between attributes and user behavior (voting or accepting an answer). Error bars indicate the variance of the best-fit values values across two to twenty answers.

We take logistic regressions for votes cast before any answer was accepted, votes after an answer was accepted, as well as choices to accept answers. The average and variance of the regression parameters across 2 to 20 answers are shown in Fig 2. Because all attributes were normalized, the larger the value, the more a change in the respective parameter correlates to a change in user behavior, relative to all others in the regression.

We find that web page order and word share have the highest regression coefficients (Fig 2). These findings alone are not necessarily surprising. We know from previous research that people’s choices are biased by the rank order of items [38–40]. Word share is potentially correlated with higher answer quality, because relatively long answers may be more informative, or they may just be easier to see (take up a large portion of screen space). We notice that both of these regression coefficients are even higher for askers than voters, across different board types, already suggesting a surprising degree of heterogeneity. Other factors, however, such as an answerer’s reputation or tenure, how thoroughly an answer is documented with hyperlinks, or how easy it is to read (readability score) do not seem to play a big role in users’ choices of which answers to vote or accept.

### 3.2 Behavior vs number of answers

**Fig 3:**
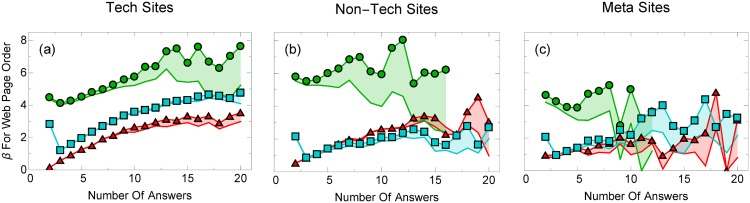
Web page order regression coefficients increase with the number of answers users see. Web page order regression coefficients for voting before (red triangles) and after (blue squares) an answer is accepted, as well as accepting an answer (green circles) for (a) technical, (b) non-technical, and (c) meta boards, with 2 to 20 answers. The shaded region represents the uncertainty in our values (see Section 2). Users increasingly depend on the web page order of an answer as the number of answers increases.

**Fig 4:**
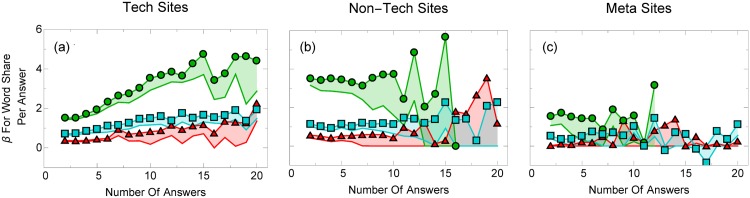
Word share regression coefficients increase with the number of answers users see. Word share regression coefficients for voting before (red triangles) and after (blue squares) an answer is accepted, as well as accepting an answer (green circles) for (a) technical, (b) non-technical, and (c) meta boards, with 2 to 20 answers. The shaded region represents the uncertainty in our values (see Section 2). Across all boards, voters appear increasingly likely to choose answers that take up a relatively large amount of web page space as the number of answers grows.

What is more surprising than the overall size of the regression coefficients, however, is that the largest coefficients, e.g., for web page order and word share, change substantially as the number of available answers to a question increases for most boards (Figs 3 and 4), and, as we will discuss shortly, the models describe the data increasingly well. Importantly, however, we see strong differences between board types. For example, the word share does not change significantly for meta boards and appears to be much smaller than for other board types.

A number of plausible explanations exist for why these coefficients depend on the number of answers:

1. Trivially, the dependence may be due to how we normalize our coefficients.
2. The subsequent answers improve upon the previous answer, or
3. Some unknown confounding variable affects both the number of answers as well as user behavior, or finally
4. User behavior changes as a function of the number of available answers.

To better understand the first hypothesis, one might object that the regression coefficient for word share, answer order, and chronological order will trivially depend on the number of answers because their normalization scheme itself depends on the number of answers. For example, if users choose longer answers over shorter ones, then, if all subsequent answers are long, the word share regression coefficients may change, even when the underlying mechanism, that users prefer longer answers, does not. To check for this effect, we incorporate both the word share and the number of words in each answer into our model. The word share attribute is found to have increasingly large regression coefficients as the number of answers grows, which suggests that the regression coefficient dependence is not trivially due to the normalization scheme. The webpage order regression coefficient, however, really does increase with the number of answers because of how the attribute is normalized. If we do not normalize the webpage order attribute, the regression coefficients would *decrease* with the number of answers. This is not an issue either, because our focus is on the relative attention that answers receive (the top answers recieve more attention than the bottom) rather than the absolute attention (that the third answer may recieve more attention when 10 answers are visible compared to when 3 answers are visible). This interpretation is similar for the chronological order attribute.

According to the second hypothesis, the last answer may be such an improvement on the previous ones that users will “flock” to it. Therefore, it should be no surprise that as the number of answers increases, changes in votes are seen. In theory, this should be captured by a significant dependence on answer’s chronological order: voters should prefer newer answers to older ones. In practice, this does not seem to be the case. The dependence on chronological order is relatively small (Fig 2), and furthermore does not change significantly with the number of answers, which is exactly the opposite of what should be expected if this hypothesis were true (S2 Fig).

The third hypothesis says that the number of answers and the behavior of the user both correlate to something else entirely; the results presented so far could be strongly affected by some confounding variable. For example, [11] finds that the reputation of later answerers on Stack Overflow, a technical board within Stack Exchange devoted to programming questions, is lower than the reputation of earlier answerers. If later voters similarly differ in reputation or some other attribute, this could potentially explain our results. We call this the “lazy voter” hypothesis, because later voters may simply be “lazier” and rely on heuristics to a greater extent. It is curious, however, that voter behavior does not seem to be significantly affected by the age of the answer, based on our regressions, and instead on the shear number of answers, as time progresses.

The last hypothesis is that users behave differently as the number of answers grows. Economics and psychologists believe that people usually do not have the time, nor inclination or cognitive resources, to process all available information, but instead, employ heuristics to quickly decide what information is important. This phenomenon, known as “bounded rationality” [27, 28], profoundly affects what information people pay attention to and the decisions they make [41]. Our results suggest that rather than thoroughly evaluating all available answers to a question on Stack Exchange, users employ cognitive heuristics to choose the “best” answer. These heuristics include choosing a top-ranked answer (Fig 3) or one that occupies more screen space (Fig 4). These heuristics become more pronounced when the volume of information (number of available answers, or cognitive load) grows.

Instead of being a cognitive heuristic, word share could plausibly reflect answer quality: high quality answers may be wordy. Interestingly, however, the regression coefficient of the number of words for each answer (rather than its share of words) is slightly negative (Fig 2), suggesting users overall prefer somewhat shorter answers if they prefer anything at all. It is intuitive that longer answers are more salient and catch a user’s eye, especially when there are many answers.

Whether the third hypothesis, or fourth, is true, our observation of a strong dependence of votes and accepts on the number of available answers suggests a strong limitation of crowdsourcing answer quality: collective judgment of quality may change with the number of answers, which is especially noticable with popular, and presumably important, questions which have many answers (Fig 1).

**Fig 5:**
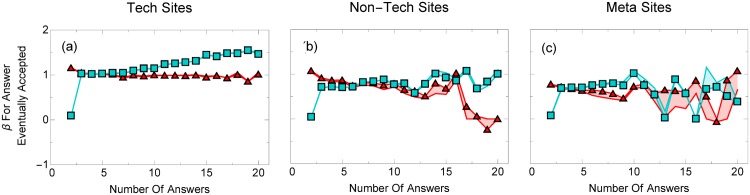
Answer acceptance increases the probability an answer will be voted on. Regression coefficients for voting on an (eventually) accepted answer before (red triangles) and after (blue squares) that answer is accepted for (a) technical, (b) non-technical, and (c) meta boards, with 2 to 20 answers. The shaded region represents the uncertainty in our values (see Section 2). There is a large and increasing vote dependence on the accepted answer once the asker accepts it, compared to before the answer is accepted, meaning the signal that this answer is accepted appears to have a statistically significant effect on voter behavior.

We see further evidence for the final two arguments in Fig 5, where we plot the regression coefficients for accepting an answer as a function of the number of answers for voters before, and after, an answer is accepted.

Voters are more likely to choose an answer that is eventually accepted (the regression coefficients are positive), but, curiously, voters are even more likely to choose the answer *after* it is accepted as the number of answers increase (the regression coefficient is usually even higher, and increases with the number of answers). In other words, although answer quality does not change before or after acceptance, users are more likely to vote on whatever the asker chooses, especially as the number of answers increase.

### 3.3 Predicting behavior

Finally, we use our trained model to predict users’ future choices of answers. After training our model on 2009-2013 data, we compared the predicted probabilities that an answer is chosen to the test set using ROC curves, in which we continuously varied the binary classifier threshold and determined from our data the true and false positive rate. Finally we take the area under the ROC curve (AUC) to determine the overall accuracy of predictions. If the AUC is near 0.5, then our model would be no better than chance. If the AUC is near 1, however, then our model predicts future behavior with a high accuracy.

**Fig 6:**
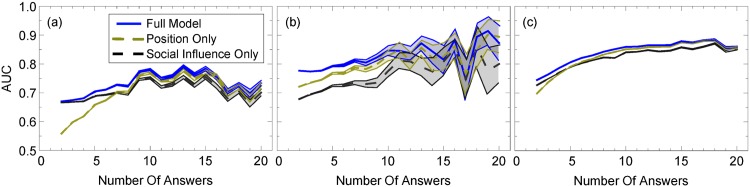
AUC versus number of answers for the full model, position null model, and social influence null model for technical boards. AUC for (a) voting before an answer is accepted, (b) accepting an answer, and (c) voting after an answer is accepted versus the number of answers in technical boards (See S4 Fig for similar plots with non-technical and meta boards). Solid lines correspond to the full models, while the lighter dashed lines correspond to the position null model, in which the probability of picking answers decreases monotonically with the web page order. Finally, the dark dashed lines correspond to the “social influence” null model, in which social signals are the only attributes used in the model. Shaded regions correspond to standard deviations in values based on bootstrapping the test data.

We find that our model predicts which answer will be chosen with increasing accuracy as the number of answers increases (Fig 6), and approaches values of 0.9 or greater (greatly beating chance, in which the AUC is 0.5). We compare our full model to two null models: a position-based null model, in which the answer order is the sole determinant of which answer will be accepted or voted on, and a social influence null model, in which answer attributes might influence users due to social cues, namely answer score, whether the answer was accepted, and the answerer’s reputation (which users can see before they upvote an answer). Interestingly, our model initially outperforms the position-based null model but with enough answers, they are virtually indistinguishable, which suggests that the answer order is the dominant attribute in our model to predict user behavior. In comparison, the social signal-based null model tends to perform poorer than both models in this range, which suggests that social signals cannot describe user behavior as well as position-based heuristics.

**Fig 7:**
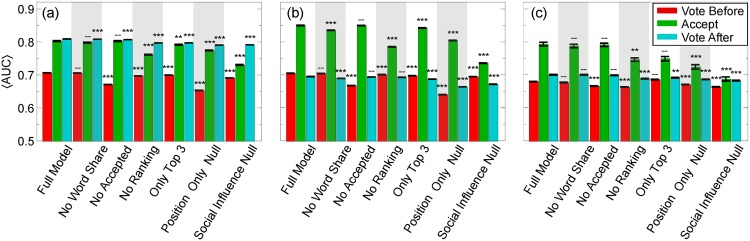
Mean AUC versus models with various attributes removed. The AUC for voting before (red lines) and after (blue lines) an answer is accepted, as well as accepting an answer (green lines) averaged over two through twenty answers, weighted by the amount of test data, in (a) technical, (b) non-technical, and (c) meta boards. See main text for descriptions of models. Error bars are standard deviations of the mean values. We remove attributes and measure the drop in mean AUC to determine the relative importance of various attributes. The statistical significance of the drop in AUC values compared to the full model is as follows: “***”, p<0.001; “**”, p<0.0028 (Bonferroni correction for 18 variables with *α* = 0.05 [42]); “–”, not significant.

To determine the overall performance of the model, we found the mean AUC weighed by the number of votes, when two to twenty answers are visible (Fig 7). We notice that the full model has the overall highest AUC, and the greatest decrease in AUC is when we remove the web page order (“No Ranking”), or whether the answer will eventually be accepted (“No Accepted”), in agreement with expectations, due to their especially high regression coefficients, although, interestingly, removing the word share had little effect for tehnical and meta boards (“No Word Share”). We find that most drops in the AUC values are statistically significant (p<0.001). Furthermore, taking only the highest coefficient attributes, web page order, whether the answer will be accepted, and word share, we find AUC values close to the full model (“Only Top 3”), which agrees with our intuition that these are the major contributing factors to our model’s behavior. As we expected, the null models performed the worst (“Position Only Null” and “Social Influence Null”), although at different points either model performed worse than the other, making conclusions about overall performance difficult. We also notice, however, differences across board types, especially in technical boards, where votes after acceptance are significantly more predictable, regardless of the model used. This is seen in meta boards, but to a far smaller degree, suggesting that something unique about technical boards may contribute to this effect. We surmise that accepting an answer significantly changes voter behavior: users default to the accepted answer, simply because it is already known to answer the question.

We also notice that askers are significantly more predictable than voters before acceptance (and usually after acceptance). We believe there are two possible reasons for this observation. First, question askers and voters look for very different answer properties. Voters may look for informative and general answers (which may be considered of higher quality), while askers look for answers that can simply solve their problem. They are, therefore, less concerned about the underlying quality so long as it is right. In addition, voters need a reputation above 15 in order to vote, which becomes a barrier to entry: typically, users must have provided answers or questions in the past that others upvoted in order to be able to vote. Askers on the other hand have no such reputation requirement. Askers might, therefore, be less able to recognize the highest quality answer, and instead rely on their own heuristics. In the past, accepted answers have been used as a gold standard of answer quality [13–15], but, if askers strongly rely on heuristics like an answer’s rank order, this puts into question whether accepted answers are the best standard. Instead, we find that the most upvoted answers may be a better standard, because voters appear to depend less on heuristics.

## 4 Discussion

We analyzed user activity over a five year period on 250 Q&A communities on the Stack Exchange network. The goal of our study was to understand the factors associated with users’ decisions of what answers to vote for or accept. Our analysis of voter and asker behavior suggests that Stack Exchange users rely on simple cognitive heuristics to choose an answer to vote for or accept, especially as the number of answers available to a question increases. First, model parameters describing the dependence of behavior on answer’s web page order and word share increase with the number of available answers. Such dependence would not necessarily exist if web page order and word share were merely proxies for answer quality. Second, based on the AUC, askers rely more on heuristics than voters do before acceptance. This suggests that answer acceptance might not be the best proxy of answer quality. Finally, voters are more likely to vote for an answer after it has been accepted than before that very same answer is accepted, especially as the number of available answers grows. Not only does acceptance appear to change a user’s judgment of answer quality, the bias appears to increase with the number of answers (i.e., cognitive load).

The behaviors we describe are consistent with, but not proof of, bounded rationality, in which decision-makers employ cognitive heuristics to make quick decisions instead of evaluating all available information [41, 43]. Moreover, people tend to use heuristics to cope with the “cognitive strain” of information overload [44]. Psychologists and behavioral scientists have identified a wide array of cognitive heuristics, which introduces predictable biases into human behavior. Social influence, *aka* “bandwagon effect”, is one such heuristic: people pay attention to the choices of others [9]. Another important heuristic for online activity is “position bias” [45]: people pay more attention to items at the top of the list or the screen than those below [38]. We find that the bandwagon effect is not necessarily as significant as position bias (rank order) in Stack Exchange (Fig 6 and S4 Fig), because the AUC in the social influence null model appears lower than the web page order-based null model as the number of answers increases, in agreement with previous studies which show that it plays a large role in user choices, even after accounting for item quality [38, 46]. That said, the overall mean AUC (Fig 7) was sometimes higher for the social influence model and at other times, for the rank-based model, making conclusions about overall performance difficult.

No matter which explanation holds, however, our work offers a cautionary note to designers of crowdsourcing systems, such as Stack Exchange: collective judgments about content quality are not necessarily accurate. To partly address this problem, the order in which answers are presented to users could be randomized until a sufficient number of votes can distinguish the quality of each answer.

Our work makes a number of methodological contributions valuable to the Data Science community. First, we use CDF normalization to make all variables commensurate, and reduce the effect of outliers. While this is a nonlinear transformation, it accounts for the distribution of variable values in the dataset, which reduces the influence of outliers and allows for fair comparisons of heterogeneous variables. Next, we handled behavioral heterogeneity by splitting data into board types and by the number of answers users see. To check robustness of regression results, we used two types of penalized regression and left out the largest Q&A board for each board type from our trained dataset. Finally, we measured the uncertainty in parameter coefficients using CV error estimates. This is the only method we are aware of to produce errors in penalized regression parameters, and the uncertainties from our regressions appear reasonable.

Our analysis of observational data cannot completely control for known and unknown covariates. For example, we cannot completely separate the effects of cognitive heuristics from those of answer quality. A necessary step in future research is to conduct a laboratory study to control for variation in answer quality, similar to previous studies [38, 46], to quantify the degree to which crowds are “myopic.” Despite known limitations, our work highlights the benefits of using data mining to understand and predict human behaviors, and may provide insight into improving the quality and performance of crowdsourcing systems.

## Supporting information

S1 Fig Changing the penalized regression method does not qualitatively change our findings. Ridge regression coefficients for voting on an (eventually) accepted answer before (red triangles) and after (blue squares) that answer is accepted, as well as accepting an answer (green circles) for (left column) technical, (central column) non-technical, and (right column) meta boards, with 2 to 20 answers. The shaded region represents the uncertainty in our values (see Section 2). We see that using a different penalized regression does not significantly affect our results (compare to Figs 3–5). (a-c) fits to the web page order attribute, (d-f) fits to chronological order, (g-i) fits to the word share attribute, (j-l) fits to the answer eventually accepted attribute.
(EPS)

S2 Fig Removing the largest board from our fits does not qualitatively change our findings. LASSO regression coefficients for voting on an (eventually) accepted answer before (triangles) and after (squares) that answer is accepted, as well as accepting an answer (circles) for (left column) technical, (central column) non-technical, and (right column) meta boards, with 2 to 20 answers. Colors indicate fits to the entire training dataset, while white markers represent fits with the largest board removed. The shaded region represents the uncertainty in our values (see Section 2). We see that removing the largest board does not significantly affect our results. (a-c) fits to the web page order attribute, (d-f) fits to chronological order, (g-i) fits to the word share attribute, (j-l) fits to the answer eventually accepted attribute.
(EPS)

S3 FigCDF normalization is especially resiliant to data noise.The standard deviation divided by the mean of best fit coefficients of simulated data (a measure of the relative variance of parameter estimates), where values are 1 with probability $\frac{1}{1+\exp(-(ax+by))}$ and 0 otherwise, and *x* and *y* are independent variables. In these simulations, *x* is normally distributed, and *y* has the following distributions: normal, exponential (*λ* = 5), log-normal (*μ* = 3, *σ* = 3), and Pareto (*y*_*min*_ = 1, *α* = 1.5, 2.0, and 3.0), for (a) *a* = −10, *b* = −10; (b) *a* = −10, *b* = 1; (c) *a* = 1, *b* = −10; and (d) *a* = 1, *b* = 1. 1000 different sets of simulated data were created and fit using the logistic regression with *x* and *y* unnormalized, *x*, and *y* CDF normalized, and *x*, and *y* normalized by their standard deviations. We find that the standard deviation normalization produces more variance in the data than CDF normalization, which suggests that CDF normalization creates more robust parameter estimates.(EPS)

S4 Fig AUC versus number of answers for the full model, position null model, and social influence null model for non-technical and meta boards. AUC for (a) voting before an answer is accepted, (b) accepting an answer, and (c) voting after an answer is accepted versus the number of answers in non-technical boards, and (d-f) equivalent plots for meta boards. Solid lines correspond to the full models, while the lighter dashed lines correspond to the position null model, in which the probability of picking answers increases monotonically with the web page order. Finally, the dark dashed lines correspond to the “social influence” null model, in which the score, answer acceptance (when voting), and reputation (which is visible to all users) are the only attributes used in our model. Shaded regions correspond to standard deviations in values based on bootstrapping the test data.
(EPS)
